# The pandemic *Escherichia coli* sequence type 131 strain is acquired even in the absence of antibiotic exposure

**DOI:** 10.1371/journal.ppat.1008162

**Published:** 2019-12-19

**Authors:** Grant R. Whitmer, Ganga Moorthy, Mehreen Arshad

**Affiliations:** 1 Feinberg School of Medicine, Northwestern University, Chicago, Illinois, United States of America; 2 Department of Pediatrics, Duke University, Durham, North Carolina, United States of America; 3 Department of Pediatrics, Ann & Robert H. Lurie Children’s Hospital of Chicago, Chicago, Illinois, United States of America; Tufts Univ School of Medicine, UNITED STATES

## Introduction

*Escherichia coli* sequence type 131 (ST131) is a pandemic multidrug resistant (MDR) strain that has had unprecedented global expansion in the last decade and is now the predominant resistant *E*. *coli* in both the adult and pediatric populations [1]. ST131 has been associated with a variety of invasive infections, resulting in higher healthcare costs and increased morbidity and mortality. These strains produce extended-spectrum β-lactamases (ESBLs), usually encoded by large plasmids, which can severely limit treatment options, delaying adequate therapy and increasing the use of last-resort antimicrobials such as carbapenems and colistin [2]. They are most commonly associated with CTX-M-15, a broad spectrum cephalosporinase. It is estimated that overall prevalence ranges from 12%–30% of all *E*. *coli* isolates, 70%–80% of fluoroquinolone-resistant *E*. *coli* isolates, and 50%–60% of all ESBL-producing *E*. *coli* isolates [3]. Rates of asymptomatic fecal carriage in the community are increasing worldwide, though the exact bacterial or host factors involved are not fully understood. Although ST131 strains are not thought to be hypervirulent, they are rapidly transmitted between hosts and are persistent gut colonizers [4–7]. In mouse models, they can out-compete commensal *E*. *coli* to colonize the gut [8].

The population structure of ST131 has recently been defined by using whole-genome sequencing (WGS) data, either with classification into clades A, B, or C or by the *fimH* alleles. Most ST131 strains fall with the 2 subclades of C1 and C2, corresponding to presence or absence of the CTX-M-15 beta-lactamase [9]. The most common *fimH* variants are *fimH30* and *fimH22* [9].

This article provides a broad overview of the ST131 colonization and transmission patterns, associated antimicrobial resistance (AMR) plasmid types, and current knowledge about its virulence and metabolism potential.

## ST131 is adept in colonizing the gastrointestinal tract of relatively healthy people even in the absence of antibiotic exposure

Given the potential consequences and severity of bacterial infections in neonates and infants, there is an urgent need to characterize ST131 colonization patterns in this population. A prospective cohort study on 80 twins and their mothers from the United States found that over a 2-year period, 18% of children and 20% of mothers had ciprofloxacin-resistant *E*. *coli* in at least one stool sample, with 91% of these *E*. *coli* being ST131 [10]. Maternal gut colonization with ciprofloxacin-resistant *E*. *coli* at the time of delivery was found to be significantly associated with colonization in twins, suggesting that these strains are adept in perinatal transmission. Interestingly, perinatal antibiotic use in mothers or early-life antibiotic exposure in infants was not significantly associated with gut colonization. Once acquired, these ESBL-producing *E*. *coli*, including ST131, can colonize the infant gut for several months (median of 7.5 months) [11]. The impact of ST131 colonization on invasive infections in infants is not known. However, given that gut bacteria can easily translocate across the epithelial barrier to cause disease, especially in premature infants, colonization with ST131 can be postulated to increase morbidity and mortality in this age group.

Overseas travel to regions with a high prevalence of ESBL-producing *E*. *coli* also appears to be inextricably linked to colonization. A Swedish study found that 32% of previously ESBL-producing *E*. *coli*-negative travelers were positive on return from Turkey, Southeast Asia, India, and North Africa, with traveler’s diarrhea and use of antibiotics during the trip found to be positively associated with colonization upon return [12]. However, in this adult population colonization appears to be transient, with only 25% and 11% remaining colonized at 6 and 12 months, respectively, in one study [5].

## ST131 can be transmitted between people of all ages and in both the community and hospital settings

A case study found that in a 6-member household, urinary tract infections (UTIs) in 2 young children caused by an ESBL-producing ST131 *E*. *coli* strain led to colonization of every member of the household in at least 2 out of 3 sampling timepoints over 19 weeks [13]. A Spanish case-control study of index patients with community and nosocomial ST131 infections found that transmission to a household member was positively associated with the household member’s use of a proton pump inhibitor and a higher age of the index patient [14]. Among hospital contacts, being dependent for basic activities or having a urinary catheter was associated with colonization. Antibiotic use was not found to be associated with household or nosocomial transmission.

Consumption of animal products may also be a cause of ST131 colonization in humans, though the transfer of this strain to humans has not been definitively shown. A study of meat products in Arizona found that 27 of 2,452 meat samples were positive for *E*. *coli* ST131-*H22* subclone [15].

## Plasmid types associated with ST131

Plasmids are classified based on their incompatibility (Inc) group. Acquisition of ESBL gene–containing plasmids by horizontal transmission is the most common mechanism of resistance among ST131 strains. Most plasmids encoding ESBLs belong to the incompatibility type F (IncF) family [16]. These plasmids are known to also carry multiple antibiotic resistance genes, including those that produce resistance against fluoroquinolones, macrolides, and aminoglycosides, making strains that harbor these plasmids difficult to treat [17]. IncF plasmids also carry multiple nonresistance genes involved in virulence (such as those associated with iron homeostasis and enterotoxin production), fertility (such as the *traT*, which encodes a conjugal transfer protein), and toxin/antitoxin systems that actively ensure maintenance of plasmids in each bacterial generation [18].

Traditional dogma dictates that acquisition of resistance genes is associated with a fitness cost. However, ESBL-producing ST131 strains do not seem to have a fitness defect [19]. This may be due to their plasmids carrying genes important for host survival, as mentioned previously. Alternately, recent evidence suggests that plasmids harbored by ESBL-producing ST131 may also interact and regulate chromosomally encoded pathogenic factors. Schaufler and colleagues identified certain strains with improved biofilm formation and a decrease in motility in the presence of their respective ESBL-encoding plasmids [19]. The decrease in motility was speculated to be due to more efficient use of nutrients, eliminating the need to swim to the peripheral zones to access richer nutrition.

## The virulence and metabolic fitness of ST131 strains is variable and does not fully explain the success of ST131

ST131 is not a hypervirulent strain and may, in some cases, be less virulent than other known uropathogenic *E*. *coli* (UPEC) strains [20]. However, the subclone ST131-*H30Rx* is reported to have a higher virulence score, even though it is not associated with a significant impact on clinical outcomes. Based on this, it seems likely that the success of ST131 in becoming a global pandemic strain is not related to its ability to cause disease.

Multiple studies have also investigated the metabolic fitness of ST131 strains as a possible explanation for the ability of this strain to be transmitted efficiently. Gibreel and colleagues studied 300 UPEC isolates collected between 2007 and 2009. Of these, 37 were found to be ST131. Using the Vitek 2 compact Automated Expert System (AES) (bioMérieux) they conducted 47 biochemical tests that were designed to test the ability of the strains to utilize carbon sources, as well as their enzymatic activity [20]. ST131 strains were found to have a significantly higher positive test score than non-ST131 strains. Unweighted pair group method using average linkages (UPGMA) cluster analysis based on biochemical profiles showed that most ST131 clones cluster together and were closely associated with antibiotic resistance. Comparison of ST131 and commensal *E*. *coli* strains in a murine model of gut colonization also showed that ST131 was able to out-compete the latter strain and colonize the gut without inducing a significant host immune response [8].

However, work done by other research groups has not shown the same strong correlation of ST131 strains with higher metabolic activity. Alqasim and colleagues showed that among the 50 UPEC isolates they studied, of which half were ST131, metabolic activity did not cluster according to the strain type [21]. Another study on 126 *E*. *coli* isolates, of which one-third were ST131, also showed no significant difference in metabolite utilization between ST131 and non-ST131 strains [22].

## Future directions

Although the bacterial factors responsible for the successful global spread of ST131 remain an enigma, it is becoming clear that this success is largely related to its ability to successfully and persistently colonize the mammalian gut [20]. Several recent studies have therefore focused on characterizing the intestinal colonization traits of ST131. Adhesion to the intestinal epithelium plays a key role in gut colonization and is largely mediated by type 1 fimbriae and their interaction with the manosylated receptors on epithelial surfaces. The expression of fimbriae is phase mediated in most UPEC strains; however, in ST131, this expression is altered because of the insertional inactivation of the *fimB* gene [23]. This mechanism is also important for adherence and invasion into the uroepithelium and colonization of the mouse bladder in an animal model of UTI [24].

Ongoing investigations have focused on the role of mannosides in decreasing gut colonization with ST131 [6]. Another strategy currently being explored includes the use of UPEC-specific bacteriophages to reduce the colonization burden of these strains without effecting normal commensals [25].

## Conclusion

The rapid spread of MDR *E*. *coli* ST131 across the world has created new challenges in the treatment of common community-acquired infections such as UTIs, in addition to increasing healthcare cost, morbidity, and mortality in hospitalized patients. Its ability to be transmitted between hosts and persistently colonize the gut even in the absence of antibiotic exposure suggests that traditional antimicrobial stewardship practices will not be sufficient to curb its spread. Understanding the bacterial and host factors involved in this phenomenon is a critical first step to limiting the expansion of ST131.

## References

[1] Brolund A, Edquist PJ, Makitalo B, Olsson-Liljequist B, Soderblom T, Wisell KT, et al Epidemiology of extended-spectrum beta-lactamase-producing *Escherichia coli* in Sweden 2007–2011. Clin Microbiol Infect. 2014;20(6):O344–52. Epub 2013/10/15. 10.1111/1469-0691.12413 . 24118431

[2] Johnson JR, Johnston B, Clabots C, Kuskowski MA, Castanheira M. *Escherichia coli* sequence type ST131 as the major cause of serious multidrug-resistant *E*. *coli* infections in the United States. Clin Infect Dis. 2010;51(3):286–94. Epub 2010/06/25. 10.1086/653932 . 20572763

[3] Banerjee R, Johnson JR. A new clone sweeps clean: the enigmatic emergence of *Escherichia coli* sequence type 131. Antimicrob Agents Chemother. 2014;58(9):4997–5004. Epub 2014/05/29. 10.1128/AAC.02824-14 24867985 PMC4135879

[4] Hilty M, Betsch BY, Bogli-Stuber K, Heiniger N, Stadler M, Kuffer M, et al Transmission dynamics of extended-spectrum beta-lactamase-producing Enterobacteriaceae in the tertiary care hospital and the household setting. Clin Infect Dis. 2012;55(7):967–75. 10.1093/cid/cis581 22718774 PMC3436924

[5] OstholmBalkhed A, Tarnberg M, Nilsson M, Nilsson LE, Hanberger H, Hallgren A, et al Duration of travel-associated faecal colonisation with ESBL-producing Enterobacteriaceae—A one year follow-up study. PLoS ONE. 2018;13(10):e0205504 Epub 2018/10/26. 10.1371/journal.pone.0205504 30356258 PMC6200250

[6] Sarkar S, Hutton ML, Vagenas D, Ruter R, Schuller S, Lyras D, et al Intestinal colonization traits of pandemic multidrug-resistant *Escherichia coli* ST131. J Infect Dis. 2018;218(6):979–90. Epub 2018/02/23. 10.1093/infdis/jiy031 29471349 PMC6093498

[7] Lavigne JP, Vergunst AC, Goret L, Sotto A, Combescure C, Blanco J, et al Virulence potential and genomic mapping of the worldwide clone *Escherichia coli* ST131. PLoS ONE. 2012;7(3):e34294 10.1371/journal.pone.0034294 22457832 PMC3311635

[8] Vimont S, Boyd A, Bleibtreu A, Bens M, Goujon JM, Garry L, et al The CTX-M-15-producing *Escherichia coli* clone O25b: H4-ST131 has high intestine colonization and urinary tract infection abilities. PLoS ONE 2012;7(9):e46547 Epub 2012/10/03. 10.1371/journal.pone.0046547 23029548 PMC3460912

[9] Stoesser N, Sheppard AE, Pankhurst L, De Maio N, Moore CE, Sebra R, et al Evolutionary History of the Global Emergence of the *Escherichia coli* Epidemic Clone ST131. MBio. 2016;7(2):e02162–15. 10.1128/mBio.02162-15 27006459 PMC4807372

[10] Gurnee EA, Ndao IM, Johnson JR, Johnston BD, Gonzalez MD, Burnham CA, et al Gut Colonization of Healthy Children and Their Mothers With Pathogenic Ciprofloxacin-Resistant *Escherichia coli*. J Infect Dis. 2015;212(12):1862–8. Epub 2015/05/15. 10.1093/infdis/jiv278 25969564 PMC4655851

[11] Rodriguez-Revuelta MJ, Lopez-Cerero L, Serrano L, Luna-Lagares S, Pascual A, Rodriguez-Bano J. Duration of Colonization by Extended-Spectrum beta-Lactamase-Producing Enterobacteriaceae in Healthy Newborns and Associated Risk Factors: A Prospective Cohort Study. Open Forum Infect Dis. 2018;5(12):ofy312 Epub 2018/12/21. 10.1093/ofid/ofy312 30568982 PMC6293479

[12] Vading M, Kabir MH, Kalin M, Iversen A, Wiklund S, Naucler P, et al Frequent acquisition of low-virulence strains of ESBL-producing *Escherichia coli* in travellers. J Antimicrob Chemother. 2016;71(12):3548–55. Epub 2016/08/28. 10.1093/jac/dkw335 . 27566312

[13] Madigan T, Johnson JR, Clabots C, Johnston BD, Porter SB, Slater BS, et al Extensive Household Outbreak of Urinary Tract Infection and Intestinal Colonization due to Extended-Spectrum beta-Lactamase-Producing *Escherichia coli* Sequence Type 131. Clin Infect Dis. 2015;61(1):e5–12. Epub 2015/04/02. 10.1093/cid/civ273 25828998 PMC4542914

[14] Morales Barroso I, Lopez-Cerero L, Navarro MD, Gutierrez-Gutierrez B, Pascual A, Rodriguez-Bano J. Intestinal colonization due to *Escherichia coli* ST131: risk factors and prevalence. Antimicrob Resist Infect Control. 2018;7:135 Epub 2018/11/27. 10.1186/s13756-018-0427-9 30473785 PMC6238289

[15] Liu CM, Stegger M, Aziz M, Johnson TJ, Waits K, Nordstrom L, et al *Escherichia coli* ST131-*H22* as a Foodborne Uropathogen. MBio. 2018;9(4). Epub 2018/08/30. 10.1128/mBio.00470-18 30154256 PMC6113624

[16] Nicolas-Chanoine MH, Bertrand X, Madec JY. *Escherichia coli* ST131, an intriguing clonal group. Clin Microbiol Rev. 2014;27(3):543–74. Epub 2014/07/02. 10.1128/CMR.00125-13 24982321 PMC4135899

[17] Villa L, Garcia-Fernandez A, Fortini D, Carattoli A. Replicon sequence typing of IncF plasmids carrying virulence and resistance determinants. J Antimicrob Chemother. 2010;65(12):2518–29. Epub 2010/10/12. 10.1093/jac/dkq347 . 20935300

[18] Lanza VF, de Toro M, Garcillan-Barcia MP, Mora A, Blanco J, Coque TM, et al Plasmid flux in *Escherichia coli* ST131 sublineages, analyzed by plasmid constellation network (PLACNET), a new method for plasmid reconstruction from whole genome sequences. PLoS Genet. 2014;10(12):e1004766 10.1371/journal.pgen.1004766 25522143 PMC4270462

[19] Schaufler K, Semmler T, Pickard DJ, de Toro M, de la Cruz F, Wieler LH, et al Carriage of Extended-Spectrum Beta-Lactamase-Plasmids Does Not Reduce Fitness but Enhances Virulence in Some Strains of Pandemic *E. coli* Lineages. Front Microbiol. 2016;7:336 Epub 2016/03/26. 10.3389/fmicb.2016.00336 27014251 PMC4794485

[20] Gibreel TM, Dodgson AR, Cheesbrough J, Fox AJ, Bolton FJ, Upton M. Population structure, virulence potential and antibiotic susceptibility of uropathogenic *Escherichia coli* from Northwest England. J Antimicrob Chemother. 2012;67(2):346–56. Epub 2011/10/27. 10.1093/jac/dkr451 . 22028202

[21] Alqasim A, Emes R, Clark G, Newcombe J, La Ragione R, McNally A. Phenotypic microarrays suggest *Escherichia coli* ST131 is not a metabolically distinct lineage of extra-intestinal pathogenic E. coli. PLoS ONE. 2014;9(2):e88374 Epub 2014/02/12. 10.1371/journal.pone.0088374 24516644 PMC3917908

[22] Hussain A, Ranjan A, Nandanwar N, Babbar A, Jadhav S, Ahmed N. Genotypic and phenotypic profiles of *Escherichia coli* isolates belonging to clinical sequence type 131 (ST131), clinical non-ST131, and fecal non-ST131 lineages from India. Antimicrob Agents Chemother. 2014;58(12):7240–9. Epub 2014/09/24. 10.1128/AAC.03320-14 25246402 PMC4249578

[23] Sarkar S, Roberts LW, Phan MD, Tan L, Lo AW, Peters KM, et al Comprehensive analysis of type 1 fimbriae regulation in fimB-null strains from the multidrug resistant *Escherichia coli* ST131 clone. Mol Microbiol. 2016;101(6):1069–87. 10.1111/mmi.13442 . 27309594

[24] Totsika M, Beatson SA, Sarkar S, Phan MD, Petty NK, Bachmann N, et al Insights into a multidrug resistant *Escherichia coli* pathogen of the globally disseminated ST131 lineage: genome analysis and virulence mechanisms. PLoS ONE. 2011;6(10):e26578 10.1371/journal.pone.0026578 22053197 PMC3203889

[25] Galtier M, De Sordi L, Maura D, Arachchi H, Volant S, Dillies MA, et al Bacteriophages to reduce gut carriage of antibiotic resistant uropathogens with low impact on microbiota composition. Environ Microbiol. 2016;18(7):2237–45. 10.1111/1462-2920.13284 . 26971586

